# Sister Mary Joseph’s Nodule: Where Umbilicus Holds the Truth!

**DOI:** 10.7759/cureus.13091

**Published:** 2021-02-03

**Authors:** Samyak Dhruv, Shamsuddin Anwar, Abhishek Polavarapu, Meena Kashi, Sherif Andrawes

**Affiliations:** 1 Internal Medicine, Northwell Health, New York, USA; 2 Gastroenterology, Northwell Health, New York, USA; 3 Pathology, Northwell Health, New York, USA

**Keywords:** umbilical skin metastasis, invasive colon cancer, end of life and hospice care, sister mary joseph nodule

## Abstract

An umbilical metastasis from an internal visceral malignancy is defined as Sister Mary Joseph's nodule (SMJN), and, although a rare finding, it is a very poor prognostic indicator. We describe an interesting case of metastatic colon cancer with SMJN, emphasizing the significance of this classic finding.

A 64-year-old female with a history of stage IV colon cancer with palliative right hemicolectomy and left hepatectomy presented to the hospital with nausea and abdominal discomfort. A computed tomography (CT) scan of the abdomen was performed, which showed small bowel obstruction secondary to metastatic tumor mass compressing the duodenum. She refused to undergo any chemotherapy or endoscopic intervention and was eventually discharged on hospice care.

During the hospital stay an umbilical ulcerative lesion was also noted, which was violaceous, measuring 4.5 x 4 cm in size, firm in consistency with irregular borders, and tender to touch. Therefore, further history was obtained from the patient about it. Several months ago, she had developed localized swelling around the umbilicus, which gradually enlarged and ulcerated later. She eventually underwent the biopsy of that umbilical lesion, which confirmed it as SMJN with metastasis from the colonic primary. However, the patient refrained from the surgical intervention of the umbilical lesion.

SMJN presents as a palpable periumbilical metastatic mass with diameter usually not exceeding 5 cm in size. It can be variable in color from violaceous to reddish brown. Once discovered, such lesions should be worked up with biopsy and imaging studies such as CT scan of the abdomen, as the nodule may be indicative of underlying malignancy or cancer recurrence. Its presence indicates a poor prognosis, with average survival time after diagnosis of SMJN of 10 months.

## Introduction

Sister Mary Joseph's nodule (SMJN) is an umbilical metastasis from a visceral malignancy, and, although a rare finding, it is described to be an indicator of poor prognosis. The incidence of SMJN is 1%-3% in people diagnosed with intra-abdominal or pelvic malignancies [1]. The primary site of malignancy associated with SMJN is significantly different in men and women. It is very important to not miss this classic finding in the clinical practice as it is an indicator of underlying advanced malignancy. We describe an interesting case of colon cancer with SMJN to emphasize its importance as a diagnostic and prognostic finding for clinicians.

This case report was also presented as a poster at ACG 2020 P0634 (S1756): Sister Mary Joseph Nodule: Where Umbilicus Holds the Truth!

## Case presentation

A 64-year-old female with a medical history of stage IV metastatic colon cancer diagnosed in 2017 was evaluated at the hospital for suspected small bowel obstruction. At the time of initial diagnosis of cancer, she had refused palliative chemotherapy. In 2018, she was hospitalized for the first time because of gastric outlet obstruction and underwent palliative hemicolectomy and hepatectomy. The surgical pathology was positive for KRAS gene mutation, whereas the MMR gene was intact. She was started on cycles of FOLFOX (folinic acid/fluorouracil/oxaliplatin) and Avastin® (bevacizumab) at that time.

**Figure 1:**
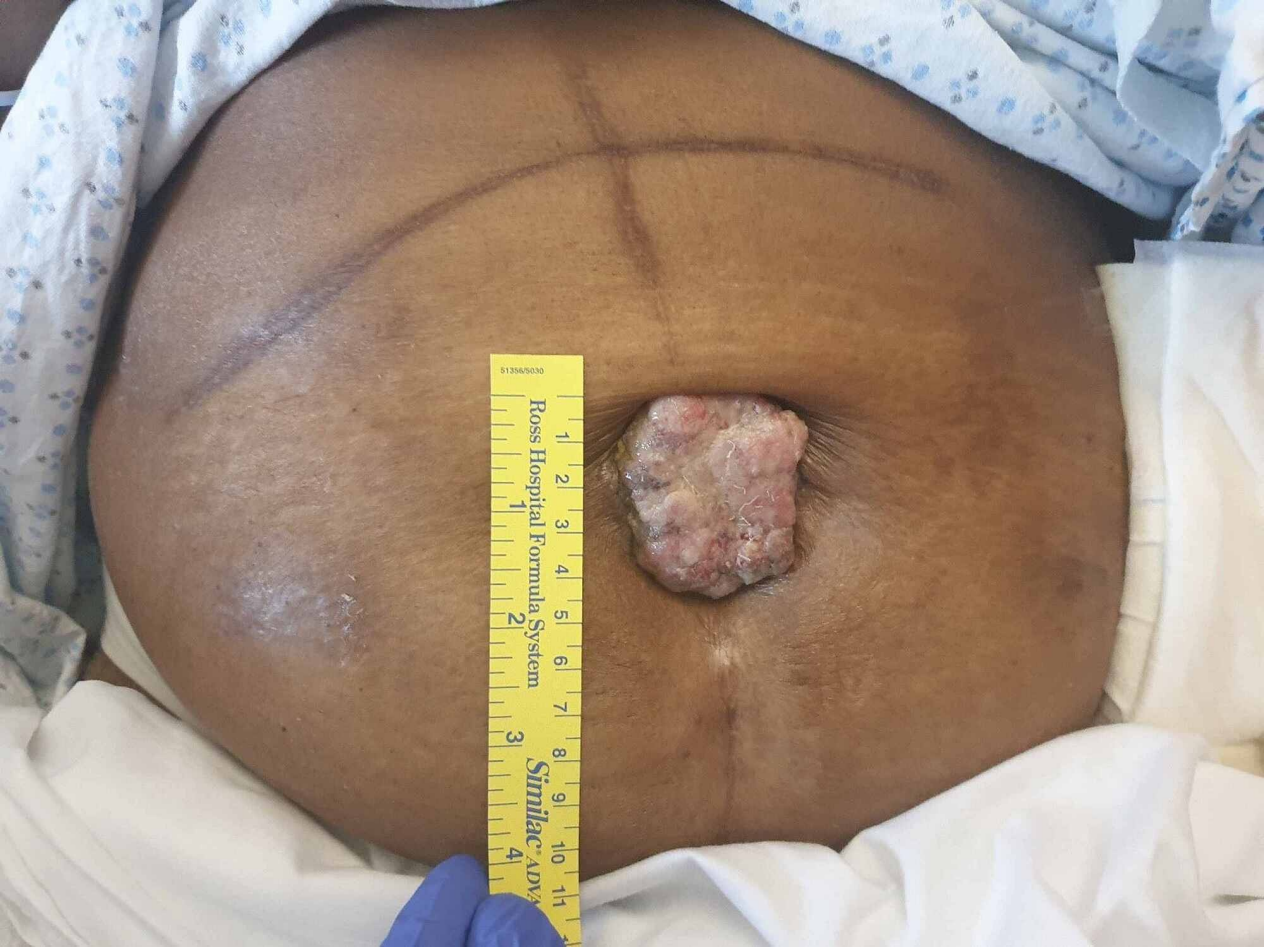
Sister Mary Joseph’s nodule, which is violaceous, measuring 4.5 x 4 cm in size with irregular borders in a patient with metastatic colon cancer.

**Figure 2:**
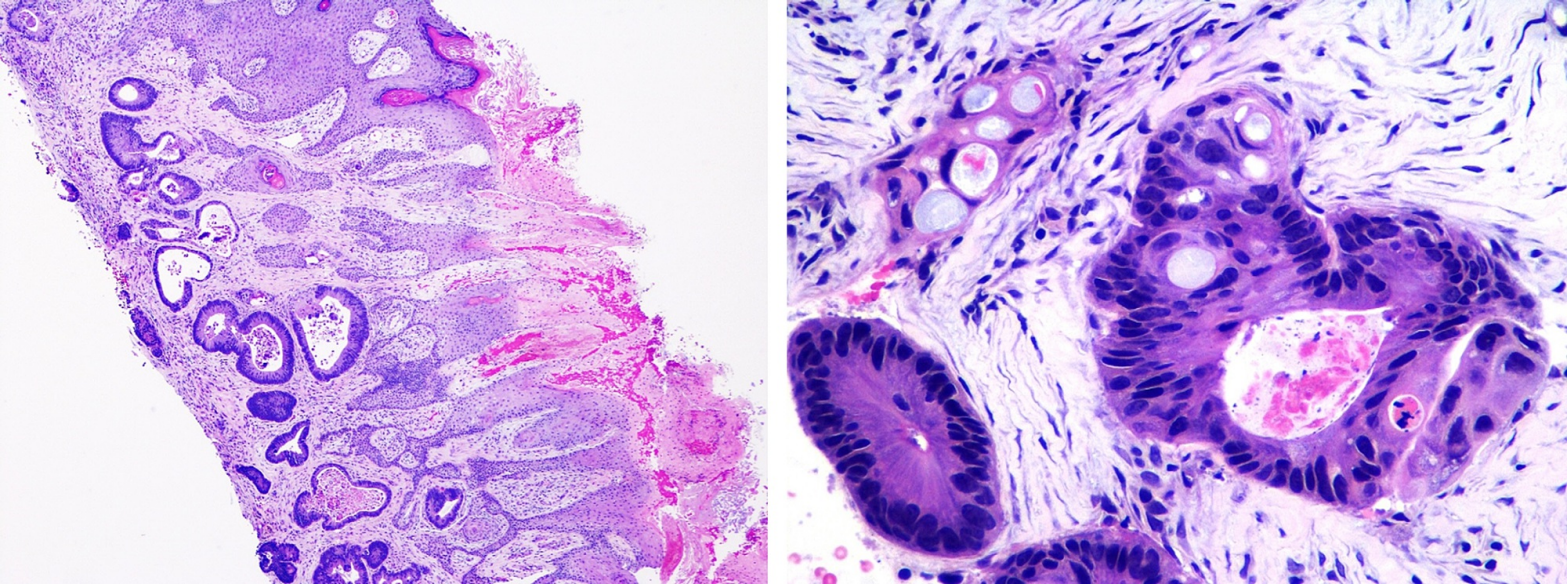
Punch biopsy of the abdominal wall showing glandular structures with comedo necrosis and clusters of carcinoma cells with signet ring cell features and surrounding desmoplastic changes consistent with infiltrating adenocarcinoma (H&E: 40X and 400X; inset: 4X and 40X).

**Figure 3:**
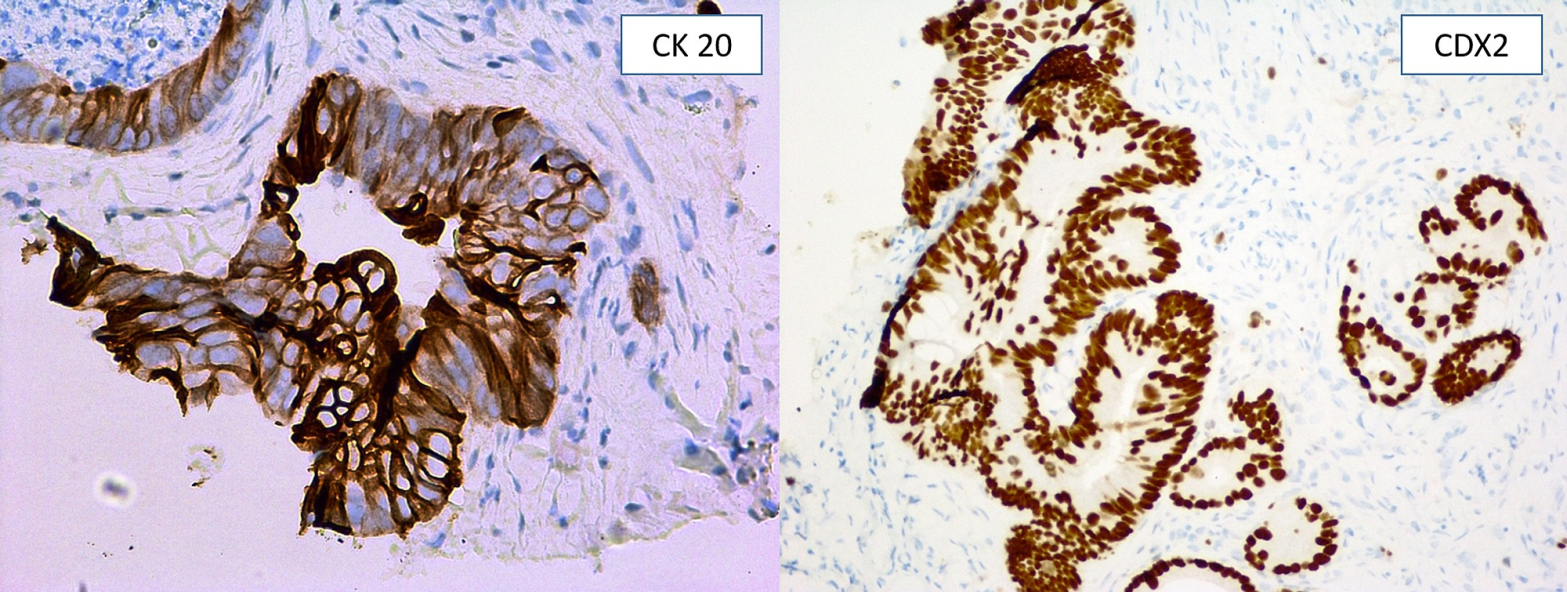
: Immunoprofile showing positivity for CK 20 AND CDX2. These findings are consistent with metastasis of the colonic primary origin.

In early 2019, she was evaluated for newly developed localized abdominal swelling around the umbilicus with ulceration (Figure 1). Imaging studies were repeated, which demonstrated progression of the disease, suggesting metastasis to the umbilicus in the form of SMJN. The chemotherapy had to be discontinued due to suspicion of superimposed infection. Besides antibiotic treatment, the patient underwent biopsy of the umbilical lesion confirming the diagnosis (Figures 2, 3). Palliative surgical resection was offered; however, the patient deferred for conservative management. She also refused to undergo any further chemotherapy.

**Figure 4:**
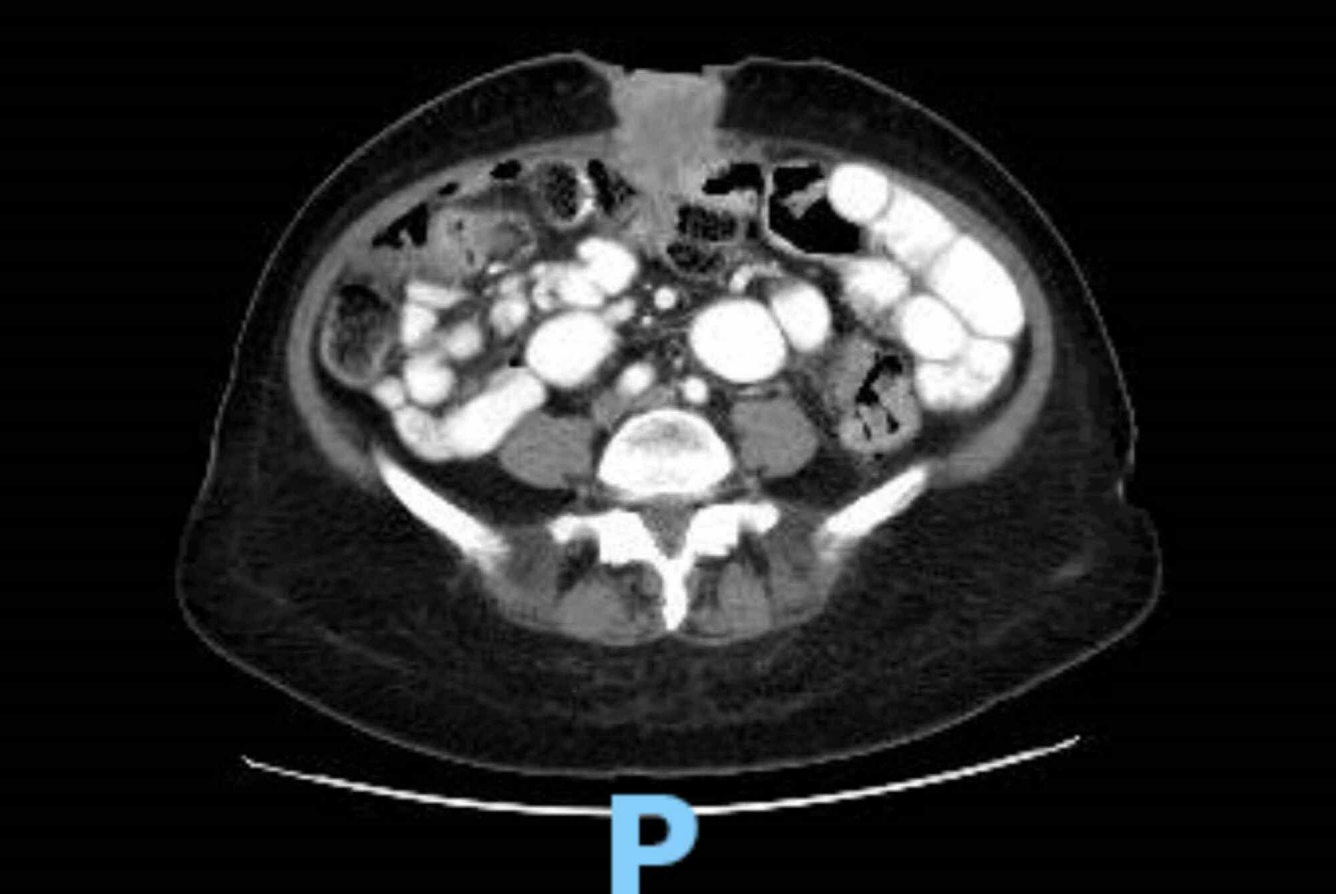
CT scan of the abdomen showing multiple metastatic abdominal wall implants along the midline anterior abdominal wall, measuring 4.1 cm in the periumbilical region and consistent with known finding of Sister Mary Joseph’s nodule in our patient.

In the current hospital admission, she presented with nausea, vomiting, bloating, and abdominal discomfort. Computed tomography (CT) of the abdomen with intravenous contrast was obtained, which demonstrated small bowel obstruction secondary to interval increase in the size of tumor burden and new hepatic metastatic lesions (Figure 4). She was initiated on intravenous fluid resuscitation and nasogastric tube suctioning for symptomatic relief; however, she refused surgical intervention and opted for hospice care. She was ultimately discharged to hospice care with comfort measures.

## Discussion

**Figure 5:**
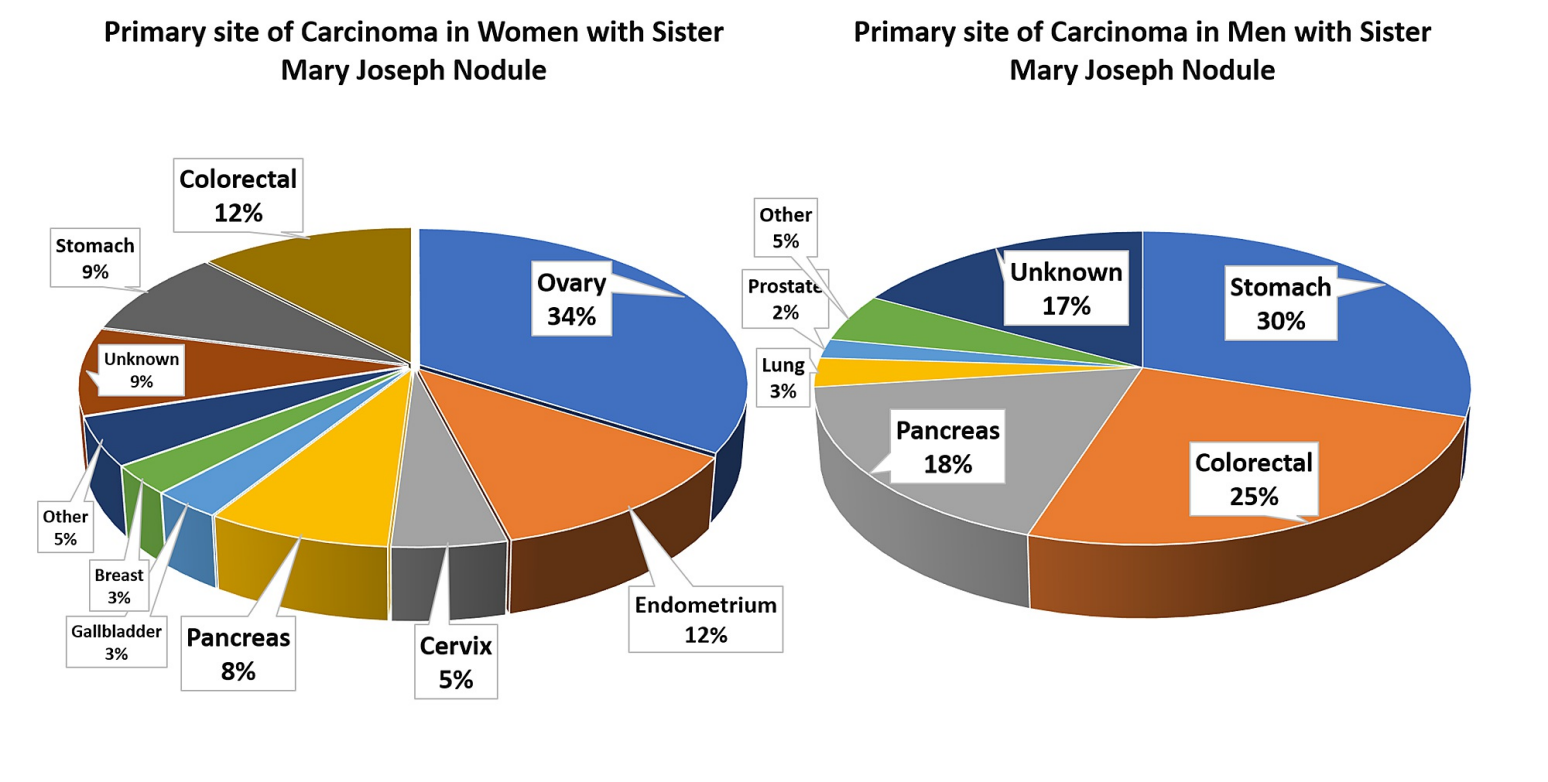
Pie chart showing the primary site of carcinoma in women and men with Sister Mary Joseph’s nodule.

Umbilical and periumbilical swellings can broadly be divided into benign and malignant lesions. The benign lesions can have broad differentials including hernia, cysts, and endometriosis. Malignant lesions can arise from primary tumors or secondarily to cancers in the abdomen and pelvis. Literature review suggests that these umbilical lesions can represent as primary tumor in 38% of the cases, endometriosis in 32%, and metastatic lesions in 30% [2]. SMJN is defined as neoplastic lesion in the periumbilical region arising as a primary tumor or representing as a site of metastasis from visceral organ malignancies such as from the gastrointestinal tract the reproductive organs. The overall incidence of SMJN is 1%-3% in general population with malignancies [1]. The primary site of malignancy associated with SMJN is significantly different in men and women. The most common primary site in men is the stomach followed by the colon and pancreas, whereas in women, the most common site is the ovary followed by endometrium, colorectal, and pancreas (Figure 5) [3].

SMJN usually presents as a palpable periumbilical nodular mass with diameter usually not exceeding 5 cm in size. It can be variable in color ranging from violaceous to reddish brown and occasionally have skin ulceration with serous or purulent discharge [4]. Umbilical nodular swelling can go unnoticed for several months before the diagnosis of malignancy. It is highly recommended that once discovered, such lesions should be worked up with biopsy and imaging studies such as ultrasound or CT of the abdomen as the nodule may be indicative of primary or secondary malignancy and cancer recurrence [5,6]. Upon discovery of umbilical lesion in our patient, she was also promptly evaluated with biopsy to determine metastasis. Biopsy is also helpful in differentiating these nodules from benign swellings in the umbilicus, such as hernia, fibroma, cyst, keloids, abscess, and benign nodules (pseudo-SMJN) [7,8].

In cases of abdominal and pelvic malignancies, several mechanisms have been defined for possible spread of metastasis to the umbilicus giving rise to SMJN. These include direct hematogenous spread or lymphatic spread of the neoplasm. On rare occasions, seeding of malignant cells may also happen to the umbilicus during surgical manipulation of abdominal viscera [9]. As SMJN tends to be indicative of disseminated malignant process, it can also be used as an indicator of poor prognosis. The average survival time reported after the diagnosis of SMJN range from few months to more than two years, with average of 10 months depending upon the etiology of primary tumor, patient's overall condition, and surgical and chemotherapeutic options available [10,11]. Therefore, early identification of SMJN can provide important clue to prompt diagnosis of underlying malignancy, enabling the patient for appropriate management and improving the survival [12].

Management of SMJN is mainly palliative. Modalities such as surgical excision and chemotherapy have proven to have limited benefit. As seen in our clinical scenario, the colon cancer had aggressively metastasized, and surgical resection was not an option. Ultimately, the patient opted for palliative care with no further aggressive measures. Given her extremely poor prognosis, she was discharged to hospice with comfort measures only [13]. SMJN is a rare physical finding but should not be missed as it can add information to the overall prognosis of the disease process.

## Conclusions

SMJN is a rare clinical finding that is described as a neoplastic mass in the periumbilical region. Its differentials include benign conditions such as cysts, hernia, endometriosis, and abscess. SMJN itself suggests underlying malignant process (primary or secondary from intra-abdominal malignancies) and warrants further investigation including biopsy and imaging studies. It can be described as an indicator of poor prognosis in malignant process with limited survival expectancy, and therefore it is important to not miss this important and classic clinical finding in a patient.
